# Waist-to-height ratio and new-onset hypertension in middle-aged and older adult females from 2011 to 2015: A 4-year follow-up retrospective cohort study from the China Health and Retirement Longitudinal Study

**DOI:** 10.3389/fpubh.2023.1122995

**Published:** 2023-02-28

**Authors:** Yang Wu, Yingmu Tong, Hai Wang, Xing Zhang, Yunxiang Long, Qinglin Li, Jie Ren, Chang Liu

**Affiliations:** ^1^Department of Hepatobiliary Surgery, The First Affiliated Hospital of Xi'an Jiaotong University, Xi'an, China; ^2^Department of General Surgery, The First Affiliated Hospital of Xi'an Jiaotong University, Xi'an, China; ^3^Department of SICU, The First Affiliated Hospital of Xi'an Jiaotong University, Xi'an, China

**Keywords:** WHtR, CHARLS, hypertension, central obesity, female

## Abstract

**Background:**

Central obesity was closely associated with hypertension. Middle-aged and older adult females, defined as those aged 45 and above, were more likely to suffer from central obesity. For waist-to-height ratio (WHtR) was used as central obesity assessment, the object of this study was to illustrate the relationship between WHtR and the incidence of hypertension in middle-aged and older adult females in China.

**Methods:**

Data used in this prospective cohort study was derived from the China Health and Retirement Longitudinal Study (CHARLS) in a baseline survey from 2011 to 2012 with a follow-up duration of 4 years. The waist-to-height ratio was calculated as waist circumstance divided by height, and the cohort was divided into different groups based on WHtR level. The outcome variable was new-onset hypertension.

**Results:**

Of the 2,438 participants included in the study, 1,821 (74.7%) had high WHtR levels (WHtR ≥ 0.5). As WHtR was closely related to new-onset hypertension in a multivariable logistics regression mode [OR: 7.89 (95% CI: 2.10–29.67)], individuals with high WHtR were also more likely to suffer from hypertension compared with low WHtR levels [OR: 1.34 (95% CI: 1.06–1.69)].

**Conclusion:**

WHtR is positively related to the risk of hypertension incidents among middle-aged and older adult females. Individuals with WHtR ≥ 0.5 were more likely to suffer from hypertension.

## Background

In recent years, the problem of global aging has continued to intensify. As individuals get older, organ function and metabolism levels decreased significantly, both of which led to metabolic-related disease. Several studies illustrated the positive relationship between aging and hypertension incidence (1). Besides these, physiological changes during menopause made a great role in regulating blood pressure (2, 3). Middle-aged and older adult females, defined as females aged 45 and above, were at high risk of suffering from hypertension.

Central obesity, manifesting as extra fat collected in the abdomen and stomach, raised attention worldwide for its rapidly increased incidence. However, the growth of age was also closely related to central obesity (4, 5). For a higher proportion of body fat and sex hormones difference, the incidence of central obesity was higher among females than males (6–8). As general obesity showed little relationship, higher relevance between central obesity and different metabolic-related diseases was illustrated (9–11). Moreover, the positive relationship between central obesity and hypertension was also revealed (12).

As middle-aged and older adult females were at high risk of suffering from hypertension, which often led to a bad outcome. There was an urgent need for risk evaluation. Waist-to-height ratio (WHtR), a proxy index for central obesity assessment, has been widely accepted as a valuable tool for a health assessment with a cut-off point of 0.5 (13). Though there were some investigations that revealed the relationship between WHtR and the incidence of metabolism-related disease (14–16), few studies explored the association between WHtR and the incidence of hypertension among middle-aged and older adult females in China. Therefore, in this study, we aimed to explore the relationship between WHtR and the incidence of hypertension and testing the usefulness of the cut-off points for health assessment in WHtR.

## Methods

### Study design and population

The cohort of this study originated from the China Health and Retirement Longitudinal Study (CHARLS) from 2011 to 2015, which is in charge of the National Development Institute of Peking University. CHARLS is an ongoing representative survey targeting individuals aged 45 and above from 450 villages and 150 counties or districts within 28 provinces in mainland China. The baseline wave was conducted between June 2011 and March 2012 and 17,708 individuals were involved. Among all the participants, 13,013 provided venous blood. All the participants were followed every 2 years. Previous research papers (17) have shown information about this.

**Figure 1:**
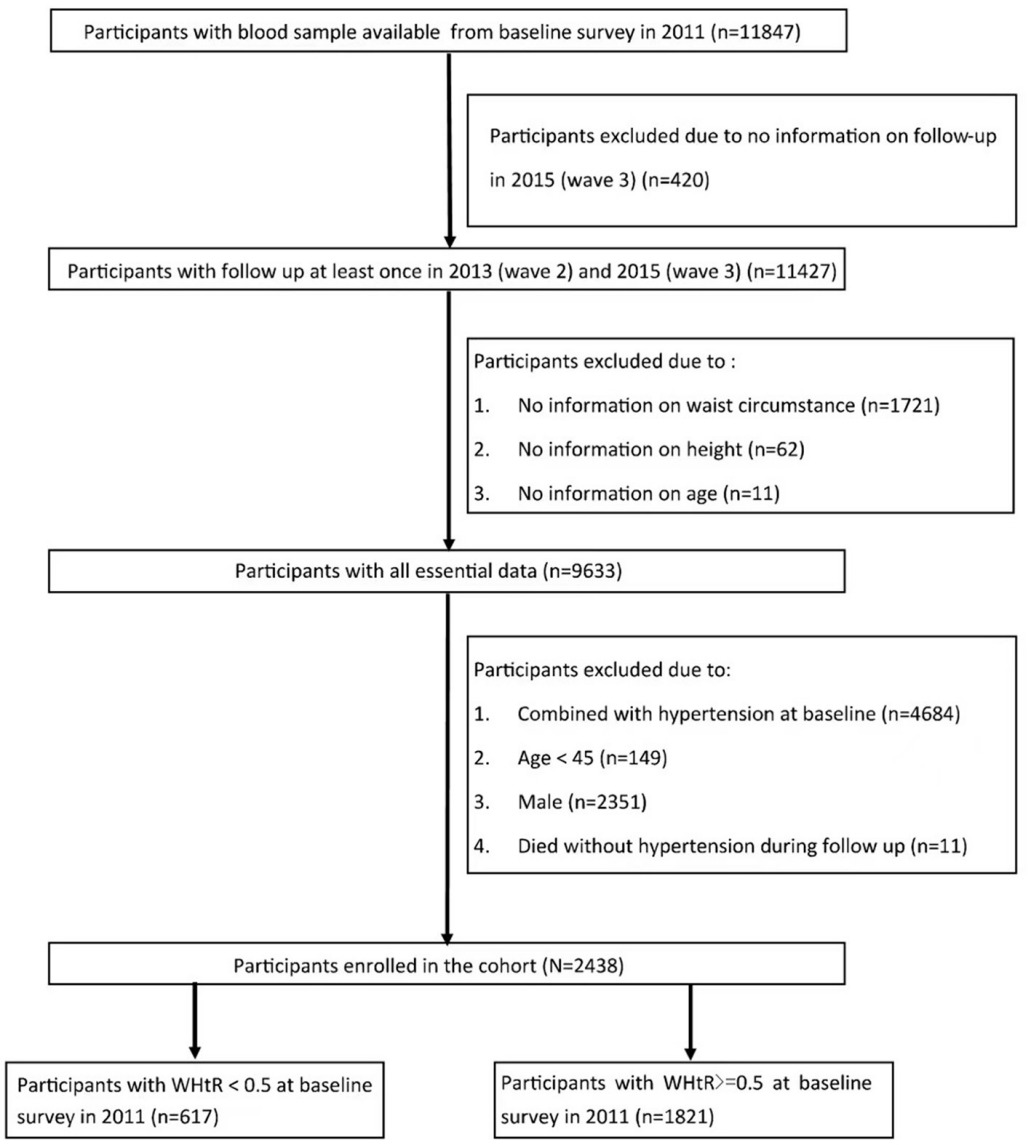
Flow chart of the study.

Individuals: (1) with complete information of venous blood sample in wave 1; (2) followed up at least once in wave 2, 3; (3) with complete information on WHtR met inclusion criteria. Individuals: (1) who were male; (2) combined with hypertension in wave 1; (3) with missing data on age or age <45; (4) who were not interviewed in 2015 were excluded from the study. A total of 2,438 individuals were enrolled in the study. As WHtR = 0.5 was used as a cut-off point for health assessment in the previous study, the cohort was divided into two groups based on WHtR level (Figure 1).

### Follow-up duration and new-onset hypertension

As all the baseline characteristic was collected from 2011 to 2012 in wave 1, all the participants followed two waves every 2 years (wave 2 and 3) until 2015. During the follow-up in waves 2 and 3, new-onset hypertension was assessed by the following criteria: (1) an SBP higher than 140 mm Hg or a DBP higher than 90 mmHg; (2) self-report of a doctor diagnosis; and (3) self-report of antihypertensive treatment.

### Other covariates

The interviewers trained by CHARLS collected information on demographic background, health status, and biomarkers according to the questionnaire. Demographic background including age, gender (male/female), education level (illiteracy, primary school, middle school, high school, and above), residence (urban/rural), marital status (married/single) were recorded, Health status consisting of 14 comorbidities (hypertension, diabetes, dyslipidemia, cancer, kidney disease, stroke, heart problem, liver disease, chronic lung disease, digestive disease, nervous problem, memory-related diseases, arthritis, and asthma) and comorbidity-related treatment taken by respondents. The options used to assess the history of alcohol drinking during the interview included: (1) I never had a drink; (2) I used to drink less than once a month; and (3) I used to drink more than once a month. In our study, option 2 and option 3 were regarded as a history of alcohol drinking. Biomarkers, including weight, height, waist circumstance, systolic pressure, and diastolic pressure, were all tested standardly by the interviewer. The blood collection, transported at 4°C temperature and sent to the local laboratory, was executed by the staff of the Chinese Center for Disease Control and Prevention (China CDC) during baseline survey. Then the plasma and buff coat were both frozen at −20°C, transported to Beijing within 2 weeks, and they would be placed in a deep freezer and stored at −80°C until assay before all the serum markers were assayed. eGFR was calculated using the CKD-EPI creatinine formula.

WHtR was calculated as waist circumstance divided by height. Height was measured by the height measuring instrument vertically. Training surveyors circled a soft tape at the navel level to measure waist circumstance. Diabetes was diagnosed as one of the following criteria: (1) self-report of a diagnosis by a doctor; (2) HbA1c ≥ 6.5%; (3) plasma glucose ≥ 11.1 mmol/L (casual) or plasma glucose ≥ 7.0 mmol/L (fasting); (4) self-report of the diabetes-related treatment. Dyslipidemia was diagnosed as one of the following criteria: (1) self-report of a diagnosis by a doctor; (2) total cholesterol (TC) ≥ 240 mg/dl; (3) high-density lipoprotein cholesterol (HDL) ≤ 40 mg/dl; (4) low-density lipoprotein cholesterol (LDL) ≥ 160 mg/dl; (5) triglycerides (TG) ≥ 150 mg/dl; (6) self-report of the anti-dyslipidemia treatment. Kidney disease was diagnosed as one of the following criteria: (1) self-report of a diagnosis by a doctor; (2) self-report of the kidney disease-related treatment; (3) Estimated glomerular filtration rate (eGFR) <60 ml/min/1.73 m^2^.

### Statistical analysis

All variables were shown as follows: continuous variables with median (IQR) and counts percentages for categorical variables. Mann-Whitney U and Chi-squared tests were used to compare baseline characteristics among cohorts with different levels of WHtR. Univariable and multivariable logistics regression was used to estimate the relationship between WHtR and new-onset hypertension. Four models were constructed, including model 1 (crude), model 2 (adjusted for age), model 3 (adjusted by age, SBP, DBP, residence, education level, digestive disease, smoking) and model 4 (adjusted by age, SBP, DBP, residence, education level, marital status, diabetes, dyslipidemia, kidney disease, cancer, chronic lung disease, liver disease, heart problem, stroke, digestive disease, nervous problems, memory-related disease, arthritis, asthma, smoking, and alcohol drinking). The interaction of different variables on new-onset hypertension was also calculated in model 4. Restrict cubic spline (RCS) functions and smooth curve fitting (penalized spline method) were used to assess the dose-response relationship and the potential non-linear relationship between WHtR and new-onset hypertension. Receiver Operating Characteristic (ROC) analyses were used to compare the effectiveness of new-onset hypertension prediction between WHtR and BMI. As the age of 60 was regarded as criterion for older people, so cut-off of age at 60 was chosen to assess the relationship between WHtR and new onset hypertension in different age groups. Both sensitivity and subgroup analysis were used to test the robustness of our findings.

Statistical analyses were performed using the R package (version 4.2.1), and *p* < 0.05 was considered statisticallysignificant.

## Results

### Baseline characteristics of study participants

There were 2,438 individuals included in the final cohort. As the baseline characteristics were shown in Table 1, the median age was 55.5 years old. Individuals with WHtR ≥ 0.5 accounted for 1,821 (74.7%) of the cohort. Compared to individuals with WHtR <0.5, individuals with WHtR ≥ 0.5 were older (56.0 vs. 55.0, *p* = 0.022), had a higher level in both systolic pressures (121.0 vs. 118.0 mmHg; *p* < 0.001) and diastolic pressure (72.0 vs. 70.0 mmHg; *p* < 0.001) at baseline. Besides these, individuals with low WHtR had a significantly lower prevalence of diabetes (7.1% vs. 13.0%, *p* < 0.001) and dyslipidemia (30.8% vs. 47.2%, *p* < 0.001).

**Table 1:** Baseline characteristic of cohort.

	**Overall**	**WHtR <0.5**	**WHtR ≥0.5**	***p***
	***n* = 2,438**	***n* = 617**	***n* = 1,821**	
Age	55.50 (49.00, 61.00)	55.00 (49.00, 60.00)	56.00 (49.00, 62.00)	0.022
WHtR	0.54 (0.50, 0.59)	0.47 (0.45, 0.49)	0.56 (0.53, 0.60)	<0.001
eGFR (ml/min/1.73 m^2^)	97.71 (87.15, 104.60)	97.91 (87.72, 105.08)	97.66 (87.07, 104.45)	0.466
SBP (mmHg)	120.00 (112.00, 129.00)	118.00 (109.00, 126.75)	121.00 (112.00, 129.00)	<0.001
DBP (mmHg)	72.00 (66.00, 78.00)	70.00 (64.00, 76.00)	72.00 (66.00, 78.00)	<0.001
BMI (kg/m^2^)	23.08 (20.88, 25.42)	20.11 (18.47, 21.57)	24.11 (22.13, 26.09)	<0.001
**Residence**, ***n*** **(%)**				0.027
Urban	314 (12.9)	63 (10.3)	251 (13.8)	
Rural	2,113 (87.1)	551 (89.7)	1,562 (86.2)	
**Education level**, ***n*** **(%)**				0.04
Illiteracy	1,422 (58.3)	374 (60.6)	1,048 (57.6)	
Primary school	443 (18.2)	89 (14.4)	354 (19.4)	
Middle school	395 (16.2)	103 (16.7)	292 (16.0)	
High school and above	178 (7.3)	51 (8.3)	127 (7.0)	
**Marital status**, ***n*** **(%)**				0.925
Alone	406 (16.7)	104 (16.9)	302 (16.6)	
Married	2032 (83.3)	513 (83.1)	1,519 (83.4)	
Dyslipidemia, *n* (%)	1,049 (43.0)	190 (30.8)	859 (47.2)	<0.001
Diabetes, *n* (%)	280 (11.5)	44 (7.1)	236 (13.0)	<0.001
Cancer, *n* (%)	31 (1.3)	8 (1.3)	23 (1.3)	1
Chronic lung disease, *n* (%)	203 (8.4)	64 (10.5)	139 (7.7)	0.037
Liver disease, *n* (%)	98 (4.0)	22 (3.6)	76 (4.2)	0.588
Heart problem, *n* (%)	217 (8.9)	49 (8.0)	168 (9.3)	0.381
Stroke, *n* (%)	27 (1.1)	7 (1.1)	20 (1.1)	1
Kidney disease, *n* (%)	186 (7.6)	45 (7.3)	141 (7.7)	0.783
Digestive disease, *n* (%)	684 (28.2)	195 (31.9)	489 (27.0)	0.021
Nervous problems, *n* (%)	41 (1.7)	14 (2.3)	27 (1.5)	0.259
Memory related disease, *n* (%)	13 (0.5)	2 (0.3)	11 (0.6)	0.613
Arthritis, *n* (%)	936 (38.5)	214 (34.8)	722 (39.7)	0.034
Asthma, *n* (%)	72 (3.0)	20 (3.3)	52 (2.9)	0.728
Smoking, *n* (%)	195 (8.0)	57 (9.3)	138 (7.6)	0.217
Alcohol drinking, n (%)	174 (7.7)	47 (8.4)	127 (7.5)	0.572
Glu (mg/dl)	100.62 (93.60, 109.26)	98.82 (92.16, 107.19)	101.34 (93.96, 110.34)	<0.001
Creatinine (mg/dl)	0.67 (0.60, 0.76)	0.67 (0.60, 0.76)	0.67 (0.60, 0.76)	0.847
Total cholesterol (mg/dl)	193.30 (169.33, 217.27)	188.66 (165.66, 211.86)	195.23 (170.88, 219.59)	<0.001
Triglycerides (mg/dl)	104.43 (74.34, 146.02)	86.73 (65.49, 127.44)	108.86 (78.76, 153.10)	<0.001
HDL (mg/dl)	104.43 (74.34, 146.02)	86.73 (65.49, 127.44)	108.86 (78.76, 153.10)	<0.001
LDL (mg/dl)	117.53 (95.49, 139.95)	112.11 (93.56, 133.76)	119.07 (95.88, 141.50)	<0.001
Hb1Ac (%)	5.10 (4.90, 5.40)	5.10 (4.80, 5.30)	5.10 (4.90, 5.47)	<0.001

WHtR, waist to height ratio; eGFR, estimated glomerular filtration rate; SBP, systolic blood pressure; DBP, diastolic blood pressure; BMI, Body mass index; Glu, glucose; HDL, high density lipoprotein; LDL, low density lipoprotein; Hb1Ac, glycated hemoglobin.

### Relationship between WHtR and new-onset hypertension

**Figure 2:**
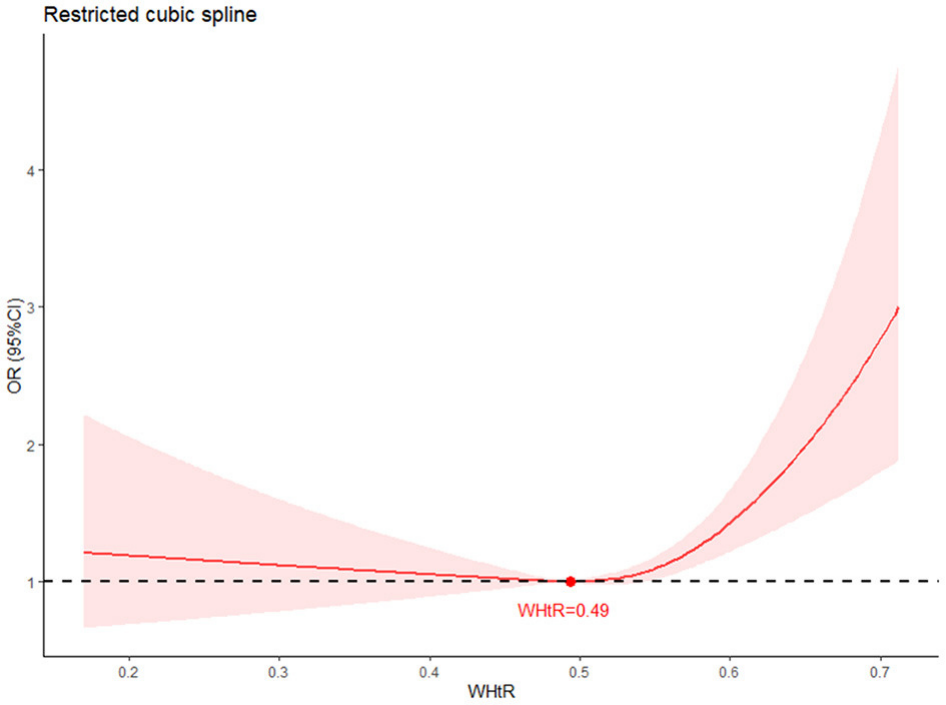
Restricted cubic spline (RCS) of the association between WHtR and new-onset hypertension. The association was adjusted for age, SBP, DBP, residence, education level, marital status, diabetes, dyslipidemia, kidney disease, cancer, chronic lung disease, liver disease, heart problem, stroke, digestive disease, nervous problems, memory related disease, arthritis, asthma, smoking and drinking. The plot showed a U-shape of WHtR and new-onset hypertension, which reached the lowest risk around 0.49 (reference). RCS, restrict cubic spline; OR, odds ratio; CI, confidence intervals.

The relationship between WHtR and new-onset hypertension was assessed in logistics regression. As the result showed, WHtR showed a positive relationship with new-onset hypertension [OR: 21.34 (95% CI: 6.49–72.93)] (Table 2) in logistics regression. The restricted cubic spline model showed a U-shape relationship between WHtR and new-onset hypertension (Figure 2) with the lowest relationship of hypertension at WHtR = 0.49.

**Table 2:** Risk factors for new-onset hypertension in univariable logistics regression.

**Characteristics**	**OR (95% CI)**	***p***
WHtR	21.34 (6.49–72.93)	<0.001
Age (per year)	1.04 (1.03–1.05)	<0.001
SBP (per mmHg)	1.06 (1.05–1.07)	<0.001
DBP (per mmHg)	1.04 (1.03–1.05)	<0.001
**Dyslipidemia**		
Without	1.00 (Ref.)	
With	1.22 (1.03–1.45)	0.024
**Education level**		
Illiteracy	1.00 (Ref.)	
Primary school	0.91 (0.72–1.14)	0.401
Middle school	0.87 (0.68–1.10)	0.244
High school and above	0.50 (0.34–0.72)	<0.001
**Digestive disease**		
Without	1.00 (Ref.)	
With	0.80 (0.66–0.97)	0.022
**Residence**		
Urban	1.00 (Ref.)	
Rural	1.43 (1.10–1.89)	0.009
**Smoking**		
Without	1.00 (Ref.)	
With	1.44 (1.06–1.94)	0.018

OR, odds ratio; CI, confidential interval; WHtR, waist to height ratio; SBP, systolic blood pressure; DBP, diastolic blood pressure.

As WHtR was used as a marker of health assessment with the cut-off point at 0.5, the relationship between different level of WHtR and new-onset hypertension were further analyzed. Different models were also used to assess the relationship between WHtR and new-onset hypertension. Individuals with WHtR ≥ 0.5 were 1.34 times higher in suffering from hypertension [OR: 1.34 (95% CI: 1.06–1.69)] (Table 3).

**Table 3:** Association between WHtR and new-onset hypertension in logistics regression.

		**Model 1**		**Model 2**		**Model 3**		**Model 4**	
		**OR (95% CI)**	***p***	**OR (95% CI)**	***p***	**OR (95% CI)**	***p***	**OR (95% CI)**	***p***
WHtR as continuous		21.34 (6.37–71.51)	<0.001	16.06 (4.82–53.51)	<0.001	6.92 (2.01–23.81)	0.002	7.89 (2.1–29.67)	0.002
WHtR as categorical	<0.5	1.00 (Ref.)		1.00 (Ref.)		1.00 (Ref.)		1.00 (Ref.)	
	≥0.5	1.55 (1.26–1.9)	<0.001	1.51 (1.23–1.86)	<0.001	1.34 (1.08–1.67)	0.009	1.34 (1.06–1.69)	0.014

WHtR, waist to height ratio; HR, hazard ratio; CI, confidential interval; SBP, systolic blood pressure; DBP, diastolic blood pressure.

Model 2: adjusted by age.

Model 3: adjusted by age, SBP, DBP, residence, education level, dyslipidemia, digestive disease, smoking.

Model 4: adjusted by age, SBP, DBP, residence, education level, marital status, diabetes, dyslipidemia, kidney disease, cancer, chronic lung disease, liver disease, heart problem, stroke, digestive disease, nervous problems, memory related disease, arthritis, asthma, smoking and alcohol drinking.

### Subgroup and sensitivity analysis

**Figure 3:**
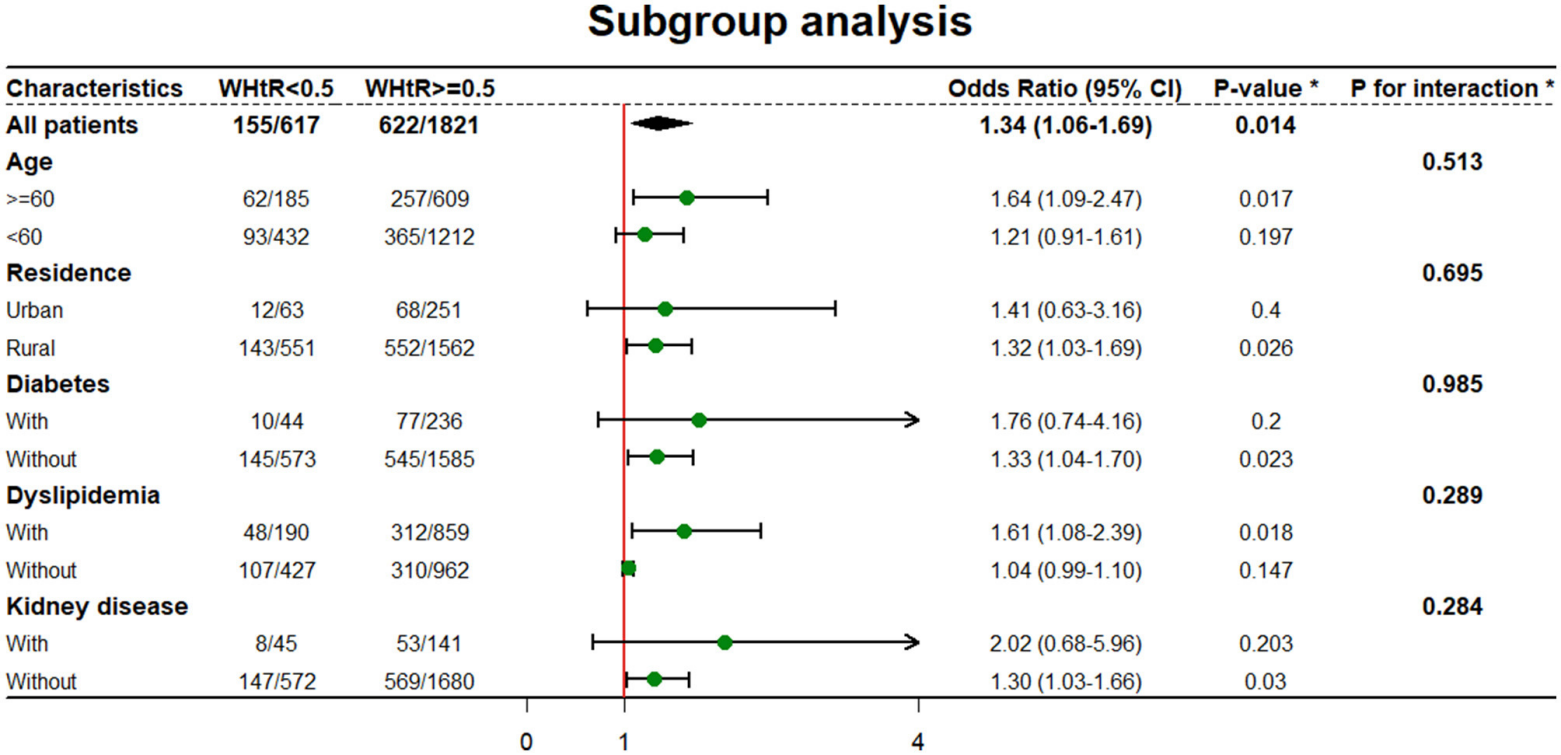
Subgroup analysis of relationship between WHtR and new-onset hypertension. *WHtR as categorical. *All model was adjusted by age, SBP, DBP, residence, education level, marital status, diabetes, dyslipidemia, kidney disease, cancer, chronic lung disease, liver disease, heart problem, stroke, digestive disease, nervous problems, memory related disease, arthritis, asthma, smoking and drinking unless the variable was used as a subgroup variable.

As is shown, participants with WHtR ≥ 0.5 were more likely to suffer from hypertension when age ≥60 [OR: 1.64 (95% CI: 1.09–2.47)], living in rural [OR: 1.32 (95% CI: 1.03–1.69)], not combined with diabetes [OR: 1.33 (95% CI: 1.04–1.70)], combined with dyslipidemia [OR: 1.61 (95% CI: 1.08–2.39)] and not combined with kidney disease [OR: 1.30 (95% CI: 1.03–1.66)] (Figure 3). WHtR was also more positively related to new-onset hypertension (Supplementary Figure 1). Sensitivity analysis was in accordance with the results (Supplementary Tables 1–3).

## Discussion

We investigated the relationship between WHtR and new-onset hypertension among middle-aged and older women in China. As the results showed, WHtR showed a positive relationship with new-onset hypertension. Besides these, the cut-off point at 0.5 was practical for health assessment. Individuals with WHtR more than 0.5 had a significantly higher incidence of hypertension when compared to others.

Though the relationship between obesity and the risk of hypertension was well-established, the mechanism of this relation was quite complex. Several mechanisms were contributing to hypertension development. As adipose tissue accumulated, the renin–angiotensin–aldosterone system (RAAS) was highly promoted, leading to high sodium and water retention (18, 19). Besides these, changes in endocrine level also played an important role (20). Decreased adiponectin secretion could also lead to insulin resistance. A high level of leptin could also result in the inflammatory response upregulating. Moreover, fatty acid accumulation was typical among obesity combined with dyslipidemia. All of these endocrine transformations could result in increased blood vessel stiffness, which is the early histology change in hypertension. Decreased estrogen levels in middle-aged and older women also played an important role in hypertension development. The level of ANP and Ang II were elevated for the reason estrogen decreased, both of which could increase the activity of RAAS (21). However, a low level of estrogen could also lead to a reduction in lipid clearance, accelerating dyslipidemia formation (22). Decreased levels of estrogen and endocrine dysfunction contributed significantly to hypertension development.

As global aging has been accelerating in recent years, age-related diseases raised more attention from all around the world. For the low level of metabolism, obesity and overweight were one of the most common comorbidities threatening the quality of life among the aged. Different obesity subtypes were further studied in recent years. The relationship between abdominal fat accumulation and increased endocrine dysfunction led to a higher incidence of cardiovascular risk among central obesity (23, 24). Besides these, central obesity was also related to a reduction in quality of life and an increment in health expenses. As the incidence of central obesity raised rapidly during recent years (7, 25), several studies indicated a higher proportion of central obesity among females than males (6, 8), which could be attributed to fertility and decreased estrogen levels. The relationship between obesity and hypertension was well-studied. Yuri et al. (26) revealed the relationship between obesity and hypertension among women in Indonesia. In a cross-section study held by Wang et al. (27), a synergistic effect of BMI and waist circumstance on the incidence of HBP (defined as SBP ≥ 140 mmHg/or DBP ≥ 90 mmHg or use of antihypertensive medication within 2 weeks) was confirmed in the aged.

Though BMI was recognized as an obesity-related marker for a long time, some investigators argued its limitation on not considering the adverse effect of intra-abdominal fat (28). WHtR was highly recommended for central obesity assessment for its easy measurement, elimination of the impact of height, and universality among different gender, and races. The effectiveness of metabolism-related disease prediction, including metabolic syndrome, hypertension, diabetes, dyslipidemia, and cardiovascular diseases, was compared among different obesity markers (29–31). According to a multicenter cross-section study held by Akbari et al. (30), WHtR performed better in hypertension prediction than WHR and BMI. Lee et al. compared the influence of different anthropometric indices on metabolic risk. WHtR was more strongly associated with hypertension in females (32). Our study used the ROC curve to assess the predicate ability and WHtR showed a higher predictive ability than BMI (Supplementary Figure 2), BMI was associated with a lower increment in new-onset hypertension when compared with WHtR (Supplementary Table 4). Besides these, our study also showed a significantly lower tendency of suffering from hypertension in WHtR <0.5 groups, which supports the usage of WHtR = 0.5 as a cut-off point for health assessment.

Obesity was closely related to the incidence of hypertension, which brought a heavy burden to public health. Our study showed a close relationship between WHtR and new-onset hypertension. Though WHtR showed a better ability for hypertension prediction than other markers among middle-aged and older females, new markers or formulas were urgently needed for much more precise prediction. Besides this, how to prevent hypertension among middle-aged and older females was also an essential factor that needs further research.

One significant advantage of our study was that this was the first study to analyze the relationship between WHtR and new-onset hypertension among middle-aged and older adult females in China. However, there were still some limitations that should be noticed. First, all the health information was collected according to self-report by participants. However, some participants might be unaware of their diseases, which could lead to bias in baseline information and the outcome variable. Second, some participants had no information about WHtR, leading to being excluded from the final cohort. These might make an impact on results.

## Conclusion

This study explored the relationship between WHtR and new-onset hypertension among middle-aged and older adult females in China. As the result shows, WHtR was positively related to hypertension. More attention should be paid to individuals with high WHtR.

## Data availability statement

The raw data supporting the conclusions of this article will be made available by the authors, without undue reservation.

## Ethics statement

The studies involving human participants were reviewed and approved by Biomedical Ethics Review Committee of Peking University (IRB00001052-11015). The patients/participants provided their written informed consent to participate in this study.

## Author contributions

YW, HW, and YT: methodology, writing, and revision. XZ, YL, QL, and JR: data curation and investigation. CL: supervision, reviewing, and editing the manuscript. All authors contributed to the article and approved the submitted version.

## Supplementary material

Supplementary Table 1 Sensitivity analysis excluded individuals combined with kidney disease for association of WHtR and new-onset hypertension in CAHRLS (2011–2015).

Supplementary Table 2 Sensitivity analysis excluded individuals combined with diabetes for association of WHtR and new-onset hypertension in CAHRLS (2011–2015).

Supplementary Table 3 Sensitivity analysis excluded individuals combined with dyslipidemia for association of WHtR and new-onset hypertension in CAHRLS (2011–2015).

Supplementary Table 4 Association between BMI and new-onset hypertension in logistics regression.

Supplementary Figure 1 Subgroup analysis of relationship between WHtR level and new-onset hypertension. ^*^WHtR as continuous; ^*^All model was adjusted by age, SBP, DBP, residence, education level, marital status, diabetes, dyslipidemia, kidney disease, cancer, chronic lung disease, liver disease, heart problem, stroke, digestive disease, nervous problems, memory related disease, arthritis, asthma, smoking and drinking unless the variable was used as a subgroup variable.

Supplementary Figure 2 ROC curves for new-onset hypertension by WHtR and BMI.
