# Supplementary material for: Waist-to-height ratio and new-onset hypertension in middle-aged and older adult females from 2011 to 2015: A 4-year follow-up retrospective cohort study from the China Health and Retirement Longitudinal Study
Source: Front Public Health. 2023 Feb 28;11:1122995. doi: 10.3389/fpubh.2023.1122995 (PMC10016226; doi:10.3389/fpubh.2023.1122995)
Supplement: Supplementary file 1 [file Data_Sheet_1.docx]

**Supplement figure legends:**

Supplement figure 1: Subgroup analysis of relationship between WHtR level and new-onset hypertension

*WHtR as continuous;

*All model was adjusted by age, SBP, DBP, residence, education level, marital status, diabetes, dyslipidemia, kidney disease, cancer, chronic lung disease, liver disease, heart problem, stroke, digestive disease, nervous problems, memory related disease, arthritis, asthma, smoking and drinking unless the variable was used as a subgroup variable

Supplement Figure 2: ROC curves for new-onset hypertension by WHtR and BMI
