# Supplementary material for: Waist-to-height ratio and new-onset hypertension in middle-aged and older adult females from 2011 to 2015: A 4-year follow-up retrospective cohort study from the China Health and Retirement Longitudinal Study
Source: Front Public Health. 2023 Feb 28;11:1122995. doi: 10.3389/fpubh.2023.1122995 (PMC10016226; doi:10.3389/fpubh.2023.1122995)
Supplement: Supplementary Table 2 — Sensitivity analysis excluded individuals combined with diabetes for association of WHtR and new-onset hypertension in CAHRLS (2011–2015). [file Table_2.docx]

| Supplement table 2: Sensitivity analysis excluded individuals combined with diabetes for association of WHtR and new-onset hypertension in CAHRLS (2011-2015) | | | | | | | | | |
| --- | --- | --- | --- | --- | --- | --- | --- | --- | --- |
|  |  | Model 1 | | Model 2 | | Model 3 | | Model 4 | |
|  |  | OR (95%CI) | P | OR (95%CI) | P | OR (95%CI) | P | OR (95%CI) | P |
| WHtR as continuous |  | 25.81  (7.09-93.95) | <0.001 | 19  (5.25-68.71) | <0.001 | 8.38  (2.22-31.62) | 0.002 | 7.45  (1.88-29.55) | 0.004 |
| WHtR as categorical | <0.5 | 1.00 (Ref.) |  | 1.00 (Ref.) |  | 1.00 (Ref.) |  | 1.00 (Ref.) |  |
|  | >=0.5 | 1.55  (1.25-1.92) | <0.001 | 1.51  (1.22-1.88) | <0.001 | 1.35  (1.07-1.7) | 0.012 | 1.33  (1.04-1.7) | 0.023 |
| Abbreviations: WHtR, waist to height ratio; OR, odds ratio; CI, confidential interval; SBP, systolic blood pressure; DBP, diastolic blood pressure; Model 2: adjusted by age; Model 3: adjusted by age, SBP, DBP, education level, dyslipidemia, digestive disease, residence and smoking; Model 4: adjusted by age, SBP, DBP ,residence, education level, marital status, dyslipidemia, cancer, chronic lung disease, liver disease, kidney disease, heart problem, stroke, digestive disease, nervous problems, memory related disease, arthritis, asthma, smoking and alcohol drinking | | | | | | | | | |
