# Supplementary material for: Waist-to-height ratio and new-onset hypertension in middle-aged and older adult females from 2011 to 2015: A 4-year follow-up retrospective cohort study from the China Health and Retirement Longitudinal Study
Source: Front Public Health. 2023 Feb 28;11:1122995. doi: 10.3389/fpubh.2023.1122995 (PMC10016226; doi:10.3389/fpubh.2023.1122995)
Supplement: Supplementary Table 4 — Association between BMI and new-onset hypertension in logistics regression. [file Table_4.docx]

| Supplement table 4: Association between BMI and new-onset hypertension in logistics regression | | | | | | | | | | | | |
| --- | --- | --- | --- | --- | --- | --- | --- | --- | --- | --- | --- | --- |
|  |  | Model 1 | |  | Model 2 | |  | Model 3 | |  | Model 4 | |
|  |  | OR (95%CI) | P |  | OR (95%CI) | P |  | OR (95%CI) | P |  | OR (95%CI) | P |
| BMI |  | 1.07 (1.04-1.09) | <0.001 |  | 1.09 (1.07-1.12) | <0.001 |  | 1.07 (1.04-1.1) | <0.001 |  | 1.08 (1.05-1.12) | <0.001 |
| Abbreviations: BMI, body mass index; OR, odds ratio; CI, confidential interval; SBP, systolic blood pressure; DBP, diastolic blood pressure  Model 2: adjusted by age; Model 3: adjusted by age, SBP, DBP, education level, dyslipidemia, digestive disease, residence and smoking; Model 4: adjusted by age, SBP, DBP ,residence, education level, marital status, diabetes, dyslipidemia, kidney disease, cancer, chronic lung disease, liver disease, heart problem, stroke, digestive disease, nervous problems, memory related disease, arthritis, asthma, smoking and alcohol drinking | | | | | | | | | | | | |
